# Correlation of Midgut Microbiota and Metabolic Syndrome-Related Lipids in Hemolymph Between Obese and Lean Silkworm Strains

**DOI:** 10.3390/insects16080798

**Published:** 2025-08-01

**Authors:** Huiduo Guo, Yalei Wang, Yu Guo, Xiangbiao Liu, Tao Gui, Mingfa Ling, Heying Qian

**Affiliations:** 1 Jiangsu Key Laboratory of Sericultural and Animal Biotechnology, School of Biotechnology, Jiangsu University of Science and Technology, Zhenjiang 212100, China; 2 Key Laboratory of Silkworm and Mulberry Genetic Improvement, Ministry of Agriculture and Rural Affairs, Sericultural Scientific Research Center, Chinese Academy of Agricultural Sciences, Zhenjiang 212100, China

**Keywords:** silkworm, metabolic syndrome, hemolymph, lipids, midgut microbiota

## Abstract

Metabolic syndrome is a global health crisis. However, there are no effective therapeutic strategies for metabolic syndrome. Therefore, this study was conducted to find out a novel silkworm-based metabolic syndrome model that bridges microbial ecology and metabolic dysregulation by integrating hemolymph lipids and midgut microbiota. Our results showed that the levels of HDL-C in the hemolymph of the lean silkworm strain were significantly higher than that in the obese silkworm strain. Furthermore, correlation analysis revealed that *Lactococcus* and *Oceanobacillus* were positively related to HDL-C levels, while *SM1A02* and *Pseudonocardia* were negatively associated with HDL-C levels. These relationships between the identified bacteria in the midgut and HDL-C, known as the “good” lipid, in the hemolymph could help guide the development of new treatments for obesity and metabolic problems like high cholesterol in humans. Overall, our results not only established a framework for understanding microbiota-driven lipid dysregulation in silkworms but also offered potential probiotic targets and a bacterial biomarker for obesity and metabolic dysfunction intervention in humans.

## Simple Summary

Metabolic syndrome presents a global health challenge lacking effective therapies. This study established a novel silkworm model integrating hemolymph lipids and midgut microbiota to investigate microbiota-driven metabolic dysregulation. We found significantly higher hemolymph HDL-C (“good” cholesterol) levels in lean versus obese silkworm strains. Critically, midgut microbiota analysis revealed that *Lactococcus* and *Oceanobacillus* positively correlated with HDL-C, while *SM1A02* and *Pseudonocardia* exhibited negative correlations. These findings define a framework for understanding microbiota–lipid interactions in silkworms and identify specific bacterial taxa as potential probiotic targets or biomarkers for developing interventions against human obesity and metabolic dysfunction.

## 1. Introduction

Metabolic syndrome, a global health crisis, is a pathologic condition characterized by abdominal obesity, hyperlipidemia, insulin resistance, and hypertension [1,2]. However, so far, there are no effective therapeutic strategies for metabolic syndrome. While rodent models have dominated metabolic syndrome research, their limitations in cost, ethical constraints, and physiological divergence from humans necessitate alternative models. The silkworm (*Bombyx mori*), a well-established lepidopteran model organism, offers unique advantages including rapid life cycles, genetic tractability, and conserved metabolic pathways, making it a promising candidate for metabolic syndrome-related studies [3,4]. Therefore, we used two distinct silkworm strains characterized by divergent body compositions, an obese silkworm strain (Dongfei) and a lean silkworm strain (Obs), in the present study. The two silkworm strains have similar long larva instars, yet they show significant body weight differences. Dyslipidemia and obesity are important manifestations of metabolic syndrome. It is currently widely believed that high triglycerides (TG), high total cholesterol (TC), and high level of low-density lipoprotein cholesterol (LDL-C) in the plasma promote the development of metabolic syndrome, while high-density lipoprotein cholesterol protects from metabolic syndrome [5,6]. Due to the close link to the lipid homeostasis and metabolic syndrome pathogenesis, the TG, TC, HDL-C, and LDL-C in the hemolymph of silkworms were quantified. We expected that these lipids would have a correlation with metabolic syndrome in insect, like in rodent models.

The gut microbiota has emerged as a pivotal regulator of lipid metabolism and metabolic syndrome through its bidirectional interactions with host metabolism [7,8]. Many studies in humans and mice have demonstrated that gut microbiota affect the absorption of nutrients from diets and have an impact on overall lipid metabolism, resulting in metabolic syndrome-related characteristics, such as obesity and hyperlipidemia [9,10,11]. Similarly, in honey bee and *C. elegans* models, the gut microbiota mediated the absorption of dietary fat, thereby influencing the host’s lipid metabolism [12,13]. In addition, a recent study revealed that midgut microbiota modulates lipid metabolism via changing the levels of certain amino acids and small molecule metabolites such as glycerophospholipids, which promoted nutrient metabolism, growth, and health of silkworms [14]. These results indicated that gut microbiota influence lipid metabolism not only in humans and mammals, but also in insects, demonstrating a certain degree of conservation in lipid metabolism signaling. In humans and rodents, dysbiosis of gut microbiota characterized by reduced microbial diversity and altered Firmicutes/Bacteroidetes (F/B) ratio is strongly associated with obesity and lipid metabolism disorders [15,16]. In the present study, we analyzed the midgut microbial communities of the midgut by 16S rDNA sequencing. Correlation analyses were performed to elucidate potential relationships between microbial composition and hemolymph lipid profiles, which may address the emerging role of gut microbiota in modulating host lipid metabolism and contribute to the treatment of metabolic syndrome.

Our study aims to find a novel silkworm-based metabolic syndrome model that bridges microbial ecology and metabolic dysregulation by integrating hemolymph lipid and midgut microbiome profiling. Our results suggested that the lean silkworm strain (Obs) has higher hemolymph TG and HDL-C levels than the obese silkworm strain (Dongfei), and the two silkworm strains show differences in composition and relative abundance of midgut microbiota. Correlation analyses indicated that *Lactococcus* and *Oceanobacillus* were positively related to HDL-C levels, while *SM1A02* and *Pseudonocardia* were negatively associated with HDL-C levels, indicating that the midgut microbiota made an interplay with hemolymph lipid metabolism. This study not only advances understanding of the lipid mechanism in silkworms but also provides a scalable platform for screening therapeutic interventions, ultimately contributing to translational metabolic syndrome research in humans.

## 2. Materials and Methods

### 2.1. Silkworm Strains and Samples

All experimental strains were provided by the Sericultural Research Institute, Chinese Academy of Agricultural Sciences (Zhenjiang, China). The larvae of the silkworm strain “Dongfei” silkworm (obese strain) and “Obs” silkworm (lean strain) were used and reared on fresh mulberry leaves at a stable temperature of 25 °C with relative humidity of 75–85%, under natural light. On day 3 of the fifth instar stage, the entire midgut and hemolymph were collected. Three larvae were dissected in each sample with 5 replicates. The samples were stored at −80 °C until used.

### 2.2. Hemolymph Biochemical Analysis

The levels of total cholesterol (TC), triglyceride (TG), low-density lipoprotein cholesterol (LDL-C), and high-density lipoprotein cholesterol (HDL-C) in hemolymph were determined using an automatic analyzer (Beckman Coulter AU5800, Brea, CA, USA) according to the manufacturer’s instructions.

### 2.3. Midgut Microbiota Analysis

Microbial DNA was extracted from the midguts using the HiPure Soil DNA Kits or HiPure Stool DNA Kits (Magen, Guangzhou, China) according to the manufacturer’s protocols. After verifying the DNA integrity, the 16S rDNA target region (the V3–V4 domain) of the ribosomal RNA gene was amplified by PCR under the following conditions: 95 °C for 5 min, followed by 30 cycles at 95 °C for 1 min, 60 °C for 1 min, and 72 °C for 1 min and a final extension at 72 °C for 7 min. The designated primers used were forward (CCTACGGGNGGCWGCAG) and reverse (GGACTACHVGGGTATCTAAT). Amplicons were evaluated with 2% agarose gels and purified using the AMPure XP Beads (Beckman, Brea, CA, USA) according to the manufacturer’s instructions. Sequencing libraries were constructed using Illumina DNA Prep Kit (Illumina, San Diego, CA, USA) in accordance with the manufacturer’s recommendation. The library quality was assessed using the ABI StepOnePlus Real-Time PCR System (Life Technologies, Foster City, CA, USA). Finally, paired-end sequencing (2 × 250 bp) was performed on the Illumina NovaSeq 6000 platform. Subsequent analyses of the 16S rDNA microbiome sequencing data, including community composition analysis, indicator species analysis, alpha diversity analysis, and beta diversity analysis, were conducted using the free online Genedenovo Cloud Platform.

### 2.4. Diversity Analysis and Differential Abundance of Taxa

Microbiome diversity analysis was performed using custom scripts developed by Genedenovo Inc. Alpha diversity indices (Sob, Chao1, Shannon, and Simpson) were calculated on the bioinformatics cloud platform (https://www.omicshare.com/, accessed on 30 August 2023). Non-metric multi-dimensional scaling (NMDS) based on unweighted unifrac and statistic analysis of Anosim test were also conducted on the cloud platform. Differential abundance of taxa between groups was assessed using the linear discriminate analysis effect size (LEfSe). The LEfSe was carried out on the cloud-based service, which includes formatting, analysis, and plotting. Cladogram and bar plots were generated to visualize the LEfSe results of differentially abundant taxa. Spearman correlation analysis was performed using R package (version 1.8.4), and heat maps were generated to illustrate the correlations between gut microbiota and obesity-related factors.

### 2.5. Statistical Analysis

Statistical analyses for lipid levels were carried out by using the SPSS 19.0 (IBM SPSS, Chicago, IL, USA). Data were presented as means ± SEM. *p*-values were calculated using a two-tailed unpaired Student’s *t*-test or the Wilcoxon rank sum test related to the indicated group, and *p* < 0.05 was considered statistically significant.

## 3. Results

### 3.1. Comparison of the Hemolymph TC, TG, LDL-C, and HDL-C Levels of the Two Silkworm Strains

**Figure 1:**
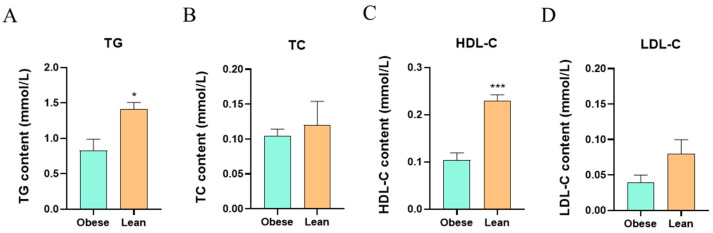
The hemolymph TG, TC, HDL-C, and LDL-C levels of the two silkworm strains. (**A**–**D**) The levels of TG, TC, HDL-C, and LDL-C in the hemolymph. Values are presented as mean ± SEM, * *p* < 0.05, and *** *p* < 0.001.

In the present study, the obese silkworm strain (Dongfei) and the lean silkworm strain (Obs) were selected for the research on the relationship between hemolymph lipid profiles and midgut microbiota. We first measured the levels of TG, TC, HDL-C, and LDL-C in the hemolymph of the two silkworm strains. Our results showed that the levels of TG and HDL-C in the hemolymph of the obese silkworm strain were significantly lower than those in the lean silkworm strain, while the levels of TC and LDL-C showed no significant difference between the two silkworm strains (Figure 1A–D). As these hemolymph lipid parameters were closely related to metabolic syndrome, our results may provide valuable reference for the establishment of an insect-based metabolic syndrome model.

### 3.2. Comparison of the Richness and Diversity of Microbiota in the Midgut of the Two Silkworm Strains

**Figure 2:**
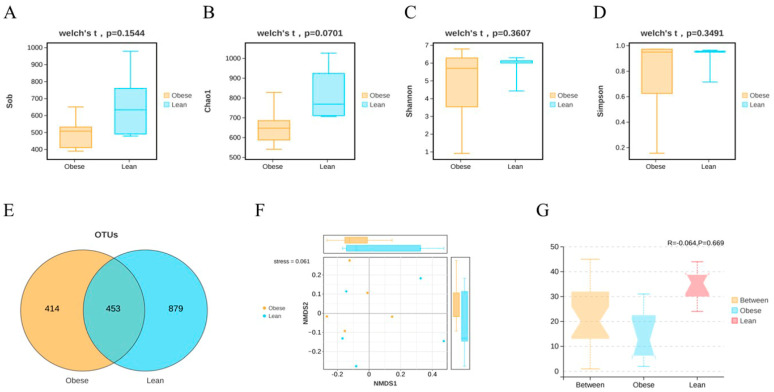
The abundance and diversity of microbiota in the midgut of the two silkworm strains. (**A**–**D**) The alpha diversity was assessed using Sob, Chao1, Shannon, and Simpson indices in the two silkworm strains. (**E**) Composition of the midgut microbiota of the two silkworm strains. (**F**) The beta diversity was evaluated through employing NMDS based on Bray–Curtis distance matrices between the two silkworm strains. (**G**) Significance of community structure differences between the two silkworm strains was tested via Anosim analysis.

Next, the midgut microbiota was classified by sequencing the v3–v4 region of the 16S rRNA gene. The Chao1 and observed-species (Sob) indices were used to evaluate microbiota richness, while the Shannon and Simpson indices provided comprehensive measures of both richness and evenness. The results showed that trends in the Sob and the Chao1 indices were generally similar between the two silkworm strains (Figure 2A,B), and the Shannon and the Simpson indices did not differ significantly either (Figure 2C,D). Although the Venn diagram shows that there are different species in the midgut of the two silkworm strains (Figure 2E), these results together suggested that there is no significant difference in α-diversity.

Based on the Bray–Curtis distance matrix for OTUs, similarities of midgut microbiota between the two silkworm strains were investigated using non-metric multi-dimensional scaling (NMDS). The NMDS plot indicated that the midgut microbiota composition of the obese silkworm strain was roughly overlapped with the lean silkworm strain, indicating the similar composition microbiota between the two silkworm strains. (Figure 2F). In addition, Anosim analysis was performed to evaluate whether group differences were significantly greater than within-group differences, with results indicating no significant differences in community structure between the two silkworm strains (R > 0, *p* > 0.05) (Figure 2G). Overall, the NMDS and Anosim analysis together reflected the highly similar community structure in midgut microbiota composition between the two silkworm strains.

### 3.3. Composition and Relative Abundance of Midgut Microbiota at Different Taxonomic Levels of the Two Silkworm Strains

**Figure 3:**
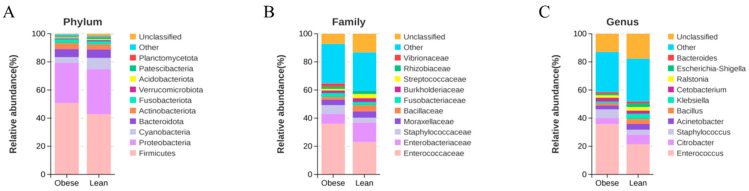
Composition and relative abundance of midgut microbiota in the two silkworm strains at different taxonomic levels. Bar graph showing relative abundance at phylum (**A**), family (**B**), and genus levels (**C**).

The composition and relative abundance of midgut microbiota in the two silkworm strains at phylum, family, and genus taxonomic levels are shown in Figure 3. At the phylum level, a total of 25 bacterial phyla were identified from the two silkworm strains, with Firmicutes (50.67% vs. 42.80%), Proteobacteria (28.64% vs. 32.00%), Cyanobacteria (4.04% vs. 8.04%), Bacteroidota (5.72% vs. 5.96%), and Actinobacteriota (4.37% vs. 3.70%) as the dominant phyla (obese vs. lean) (Figure 3A). The ratios of F/B in the obese silkworm strain and the lean silkworm strain were 8.86 and 7.17, respectively. At the family level, 133 bacterial families were identified, of which Enterococcaceae (35.99% vs. 22.97%), Enterobacteriaceae (7.03% vs. 13.76%), Staphylococcaceae (6.25% vs. 3.56%), and Moraxellaceae (3.83% vs. 4.49%) were the most prevalent (obese vs. lean) (Figure 3B). The relative abundances of Enterobacteriaceae and Erwiniaceae in the midgut of the lean silkworm strain were significantly higher than those in the obese silkworm strain (*p* < 0.05). At the genus level, 195 bacterial genera were identified, of which *Enterococcus* (35.73% vs. 21.38%), *Citrobacter* (4.28% vs. 6.80%), and *Staphylococcus* (6.24% vs. 3.56%) were dominant (obese vs. lean) (Figure 3C). The relative abundances of *Klebsiella*, *Stenotrophomonas*, *Pantoea*, and *Oceanobacillus* in the midgut of the lean silkworm strain were significantly higher than those in the obese silkworm strain (*p* < 0.05). Overall, these results suggested that the composition and relative abundance of midgut microbiota differed in the two silkworm strains at different taxonomic levels.

### 3.4. Potential Midgut Microbiota Biomarkers as Defined by LEfSe

**Figure 4:**
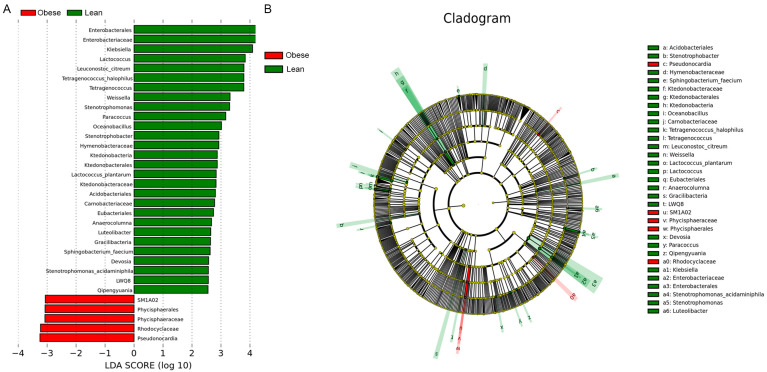
Potential midgut microbiota biomarkers as defined by LEfSe. The differentially abundant features are shown by the LDA scores (**A**) and Cladogram (**B**) between the two silkworm strains. The LDA score for discriminative features was 2.0.

Using a threshold LDA score of 2.0 for feature recognition, the relative abundance of different midgut microbiota taxa, from phylum to genus, in the two silkworm strains was evaluated using linear discriminant analysis effect size (LEfSe). As shown in Figure 4, the red bars represent the bacteria species enriched in the obese silkworm strain, while the green bars represent bacteria enriched in the lean silkworm strain. A total of 33 biomarkers were identified, with five significantly enriched in the obese silkworm strain and 28 significantly enriched in the lean silkworm strain. Specifically, two genera, *Pseudonocardia* and *SM1A02*, were significantly enriched in the obese silkworm strain, while 12 genera, including *Klebsiella*, *Lactococcus*, and *Tetragenococcus*, were significantly enriched in the lean silkworm strain. These differential bacterial communities may exert regulatory effects on the phenotypes.

### 3.5. Spearman Correlation Between Midgut Microbiota and Obesity Factors at the Genus Level

**Figure 5:**
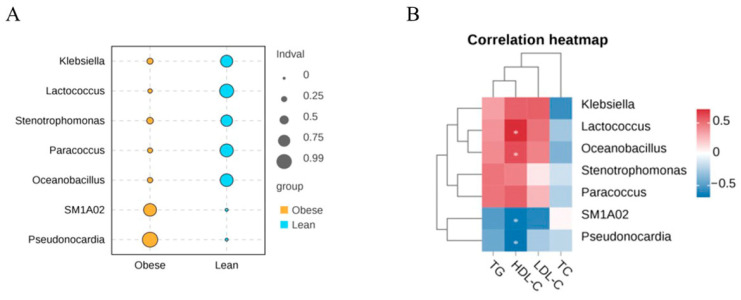
Spearman correlation between midgut microbiota and lipid profiles at the genus level. (**A**) Differential bacteria (biomarker) at genus level as determined using indicator species analysis. (**B**) Correlation between midgut microbiota and lipid profiles at the genus level. * *p* < 0.05.

Additionally, we also employed indicator species analysis to analyze the differentially abundant taxa in the midgut of the two silkworm strains at genus level. Our results suggested that the relative abundances of *Klebsiella*, *Lactococcus*, *Stenotrophomonas*, *Paracoccus*, and *Oceanobacillus* were significantly enriched in the lean silkworm strain, while *SM1A02* and *Pseudonocardia* were significantly enriched in the obese silkworm strain (*p* < 0.05) (Figure 5A). Furthermore, Spearman correlation analysis was conducted to evaluate the interplay between these midgut microbiota and lipid profiles. Our findings revealed that *Lactococcus* and *Oceanobacillus* were positively related to HDL-C levels, while *SM1A02* and *Pseudonocardia* were negatively associated with HDL-C levels, indicating that the midgut microbiota made an interplay with lipid metabolism.

## 4. Discussion

In the present study, two distinct silkworm strains, an obese silkworm strain (Dongfei) and a lean silkworm strain (Obs), were used to find out a novel silkworm-based metabolic syndrome model that bridges microbial ecology and metabolic dysregulation by integrating hemolymph lipid and midgut microbiota profiling. Previous studies in human and mice have demonstrated that dysregulation of serum lipid metabolism is one of the most important indicators of metabolic syndrome [17,18,19]. Kassahun Haile et al. reported that people who suffered from metabolic syndrome had serum lipid profile abnormalities, such as low HDL and high TG, LDL, and TC levels, compared with the normal subjects [20]. Similarly, a meta-analysis came out with the results that the most frequent metabolic syndrome components were low HDL-C and hypertriglyceridemia [21,22,23]. Additionally, a lot of studies on obesity, whether in animal models or humans, consistently revealed the decreased HDL-C and elevated TG levels in the blood [24,25,26,27,28]. On the contrary, our results showed that the level of TG in the hemolymph of the obese silkworm strain were significantly lower than that in the lean silkworm strain, while the levels of TC and LDL-C showed no significant difference between the two silkworm strains. The differences in TG levels between humans and silkworms may be related to the metabolic variations among different species. However, similar to previous studies, the levels of HDL-C in the lean silkworm strain were significantly higher than those in the obese silkworm strain, which strengthened that the HDL-C is a beneficial indicator for lipid metabolism associated with metabolic syndrome or obesity [6,29]. In addition, many studies suggested that the TG/HDL-C ratio is a superior predictor of metabolic syndrome, and a high TG/HDL-C ratio indicated a higher risk of metabolic syndrome [30,31,32]. Although there was no significant difference in the TG/HDL-C ratio between the obese silkworms and the lean ones, the TG/HDL-C ratio of the obese silkworms (8.56) was higher than that of the lean ones in our study (6.14). Collectively, our results provided valuable reference for the establishment of a silkworm-based model of metabolic syndrome to some extent.

The gut microbiota plays a significant role in maintaining the homeostasis of host health, especially during the development of metabolic diseases, such as metabolic syndrome and/or obesity in human and animal models [7,33]. In the present study, the composition and relative abundance of midgut microbiota differed in the two silkworm strains at different taxonomic levels, although trends in the richness and diversity of microbiota in the midgut of the two silkworm strains were similar. Different composition and abundance of midgut microbiota may be an important factor for the differences in hemolymph lipid levels and body weight between the two silkworm strains. Notably, the midgut microbiota of the lean silkworms was enriched with *Klebsiella*, *Lactococcus*, *Stenotrophomonas*, *Paracoccus*, and *Oceanobacillus*, whereas *SM1A02* (belonging to the *Phycisphaeraceae* family) and *Pseudonocardia* dominated in the obese strain. Similarly, it was reported that *Klebsiella* and *Lactococcus* were negatively with obesity and obesity-related metabolic disorders [28,34,35]. Furthermore, Spearman correlation analysis revealed that the *Lactococcus* and *Oceanobacillus* were positively associated with HDL-C levels, while the *SM1A02* and *Pseudonocardia* showed negative associations with HDL-C levels. Consistent with our results, *Lactococcus* seems to be a beneficial bacterium and mediated the alleviation of the non-alcoholic fatty liver disease and hyperlipidemia with restored serum HDL-C levels in rats [36,37]. In fact, numerous studies have provided evidence for an association between serum lipids and the gut microbiota [38,39,40]. Additionally, we found that *SM1A02*, a poorly characterized taxon, and *Oceanobacillus*, a marine-derived genus, may be linked to improved lipid utilization, which was enriched in lean silkworms. These results together underscored that the midgut microbiota may make contributions to lipid homeostasis in silkworm and provided promising candidates for combating obesity and related metabolic disorders.

In conclusion, our findings established a framework for understanding microbiota-driven lipid dysregulation in silkworms. The lean strain’s microbiota, enriched with HDL-C-promoting taxa, offered potential probiotic targets for obesity and related metabolic disorders intervention, while the obese-associated microbes provided biomarkers for metabolic dysfunction. However, there are some limitations on the present study, and there is a need for further investigation. (1) More factors in the hemolymph related to metabolic syndrome need to be included in the research, such as glucose, HDL, and LDL. (2) Future work should validate these interactions through microbiota transplantation, gnotobiotic silkworm establishment, and metabolic flux analysis. These comprehensive investigations will provide deeper mechanistic insights and strengthen the translational relevance of our findings regarding metabolic syndrome and obesity.

## Data Availability

The raw data supporting the conclusions of this article will be made available by the authors on request.
